# Pancreatic islet macroencapsulation using microwell porous membranes

**DOI:** 10.1038/s41598-017-09647-7

**Published:** 2017-08-23

**Authors:** Katarzyna Skrzypek, Milou Groot Nibbelink, Jéré van Lente, Mijke Buitinga, Marten A. Engelse, Eelco J. P. de Koning, Marcel Karperien, Aart van Apeldoorn, Dimitrios Stamatialis

**Affiliations:** 1 Bioartificial organs, Biomaterials Science and Technology department, MIRA Institute of Biomedical Technology and Technical Medicine, University of Twente, Enschede, The Netherlands; 2 Developmental BioEngineering, MIRA Institute of Biomedical Technology and Technical Medicine, University of Twente, Enschede, The Netherlands; 3 Department of Radiology and Nuclear Medicine, Radboud University Medical Center, Nijmegen, The Netherlands; 4 Nephrology, Leiden University Medical Center, Leiden, The Netherlands; 5 Hubrecht institute, Utrecht, The Netherlands; 6 Complex Tissue Regeneration, MERLN Institute for Technology Inspired Regenerative Medicine, Maastricht University, Maastricht, The Netherlands

## Abstract

Allogeneic islet transplantation into the liver in combination with immune suppressive drug therapy is widely regarded as a potential cure for type 1 diabetes. However, the intrahepatic system is suboptimal as the concentration of drugs and nutrients there is higher compared to pancreas, which negatively affects islet function. Islet encapsulation within semipermeable membranes is a promising strategy that allows for the islet transplantation outside the suboptimal liver portal system and provides environment, where islets can perform their endocrine function. In this study, we develop a macroencapsulation device based on thin microwell membranes. The islets are seeded in separate microwells to avoid aggregation, whereas the membrane porosity is tailored to achieve sufficient transport of nutrients, glucose and insulin. The non-degradable, microwell membranes are composed of poly (ether sulfone)/polyvinylpyrrolidone and manufactured via phase separation micro molding. Our results show that the device prevents aggregation and preserves the islet’s native morphology. Moreover, the encapsulated islets maintain their glucose responsiveness and function after 7 days of culture (stimulation index above 2 for high glucose stimulation), demonstrating the potential of this novel device for islet transplantation.

## Introduction

Type 1 diabetes is an autoimmune disorder, characterized by the specific destruction of insulin-producing β-cells within the islets of Langerhans, resulting in an absolute insulin deficiency. Currently, type 1 diabetes accounts for 5–10% of the total cases of diabetes worldwide, occurring mainly in children and young adults^1^. In fact, more than 500 000 children under 15 years of age were diagnosed with type 1 diabetes in 2015^2^. Although insulin therapy is effective in regulating the blood glucose levels, it still lacks the precise glycemic control that the normal physiological system has. Therefore, it results often in hypoglycemic events, while in the long term micro/macrovascular complications affect many patients^3^.

The replacement of β-cells by intrahepatic islet transplantation in combination with immunosuppressive drugs can restore insulin independence. However, while often successful, intrahepatic islet transplantation is associated with a high degree of islet loss, due to a multifactorial response involving an immediate blood mediated inflammatory response, auto and alloimmunity, and loss of innervation and vascularization^4^. In addition, life-long immune suppressive therapy is necessary resulting in increased risks of attacking infections or certain cancers, while the supply of high quality donor pancreas available for islet isolation and transplantation is very limited. Encapsulation using biomaterials, to provide a physical barrier between transplanted β-cells and their recipients, has emerged as a promising approach to improve transplantation outcomes eliminating the need for immunosuppression^4, 5^. Moreover, encapsulation could allow for using of not only human donor islets, but also the use of *de novo* beta cells derived from stem cells, or even xenogeneic islets and help overcome the islet donor shortage limitations.

Commonly applied strategies often focus on encapsulation of single islets using either hydrogels, or nanometer-scale coatings, such as, alginate, polyethylene glycol, polylactide-derived, or cation-anion layer by layer systems^6–8^. Although, initial results suggest that islets can maintain their function, long-term survival cannot be guaranteed. Hydrogels are, in most cases, not stable enough to support islets transplantation over long time. In recent years, the creation of alternative transplantation sites using three-dimensional scaffolds has been explored, too. Highly porous scaffolds such as poly(lactide-glycolide) sponge, vicryl or, poly(glycolic-acid) fibers meshes, with high interconnectivity have been proposed as suitable islet encapsulation devices^9–11^. Islets seeded into the macropores of these constructs can easily and quickly be provided with oxygen and the necessary nutrients. However, in most cases these constructs have large pores that permit tissue ingrowth and cell penetration. Therefore, this approach still requires the use of immunosuppressive drugs.

The main advantage of macroencapsulation technique is the control of confining the islets to one location in the body as well as the ability to retrieve the device and the possibility of islet replenishment, if necessary. However, islet encapsulation remains a difficult challenge because, by preventing cytotoxic T-lymphocyte interaction with the allogeneic beta cells, the mass transport of necessary nutrients, glucose and insulin is often compromised.

A variety of different macroencapsulation designs have been studied such as tubular chambers, sealed hollow fibers and planar devices^12^. However, poor oxygen and nutrient diffusion across the membranes was the main reason for eventual graft failure, leading to compromised islet viability. Additionally, the lack of physical separation of the islets in these macroencapsulation devices causes aggregation of the islets. This negatively affects islet structure, leading to limited diffusion of nutrients and oxygen, loss of function and apoptosis. Jiang *et al*. developed agarose hydrogel membranes with microwell patterns allowing for cells separation as a model encapsulation system^13^. Moreover, they suggested that micropatterned encapsulation systems may be able to minimize the transplantation volume, increase the encapsulation efficiency and improve the cell viability. However, the design of an immune protective macroencapsulation device for islets, should strike a balance between optimal survival of islets and shielding the same islets from the immune system.

**Figure 1:**
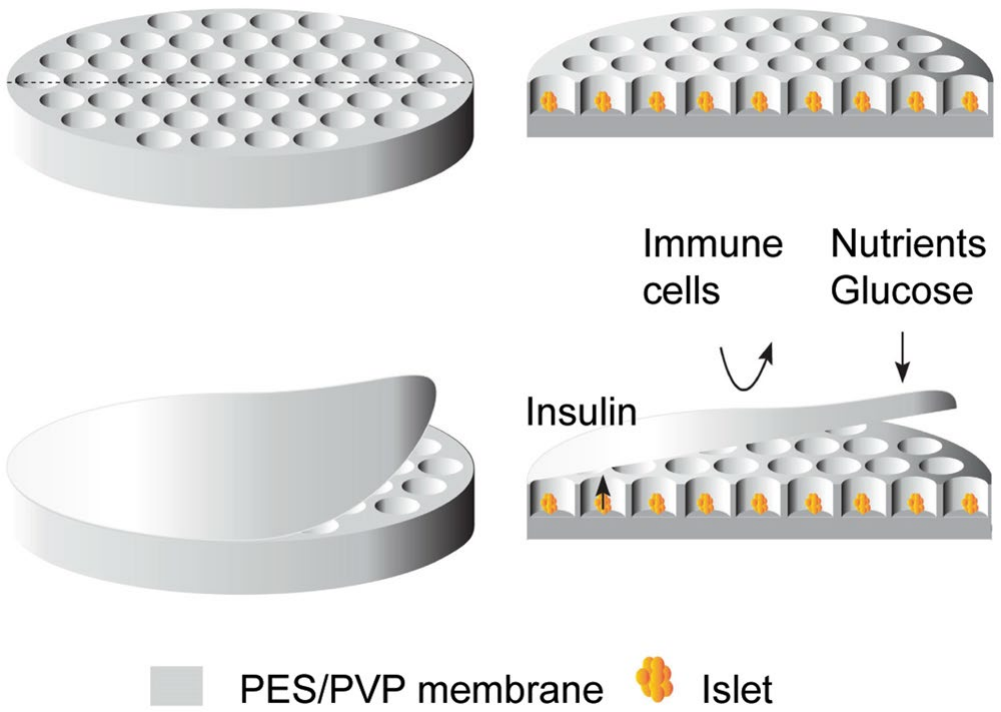
A schematic overview of the PES/PVP flat membrane encapsulation device.

In this study, we propose a novel concept for a macroencapsulation device in which islets are confined between two porous membranes. One membrane consists of microwells in which the islets are seeded and the other membrane acts as a lid, see Fig. 1. For both, the microwell and the lid membranes, the porosity is tailored to permit nutrient inflow and metabolite outflow, but protects the islets from immune cells. The microwell array allows good islet separation and prevents both spreading and aggregation, maintaining the islet’s rounded morphology.

In earlier studies, we developed open microwell scaffolds for vessel ingrowth using poly(ethylene oxide terephthalate)-poly(butylene terephthalate) (PEOT/PBT) biodegradable polymers^14^. Our aim here is to achieve a non-degradable functional closed encapsulation device. Therefore a closed porous system is developed using poly (ethersulfone) (PES)/polyvinyl pyrrolidone (PVP) polymer blend via phase separation micromolding, which is a unique method for preparation of porous microstructured membranes in one step^15–17^. PES is a non-degradable material that has high stability and good mechanical properties, and is widely used as a membrane material for blood purification and other biomedical membrane applications. Blending PES with PVP results in more hydrophilic membranes that have low fouling and, importantly for islet encapsulation, low cell adhesion properties. The porosity of the PES/PVP microwell membranes is tailored to allow insulin and glucose transport and the device performance is evaluated by analyzing the glucose responsiveness of encapsulated MIN6 mouse insulinoma cell aggregates and of human islets. Our results indicate that the PES/PVP microwell membrane, as a crucial part of macroencapsulation device, is a potential carrier for extrahepatic islet transplantation.

## Results

### Microwell structured membranes fabrication and characterization

**Figure 2:**
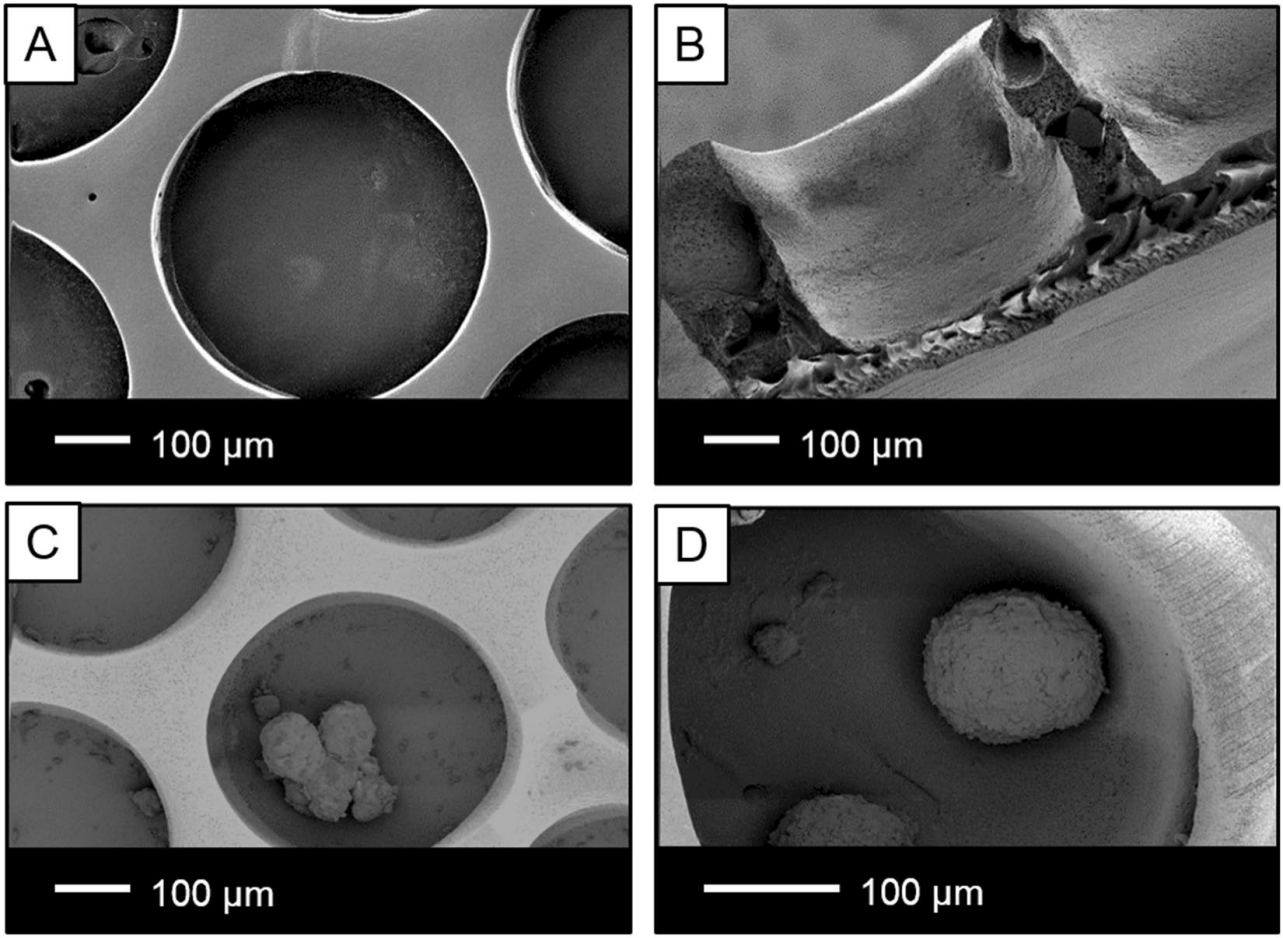
Scanning electron microscopy images of microwell membranes. (**A**) Top view, (**B**) Cross section, (**C**) Human islets inside the well, (**D**) MIN6 aggregates inside the well.

We used phase separation micromolding to fabricate uniform, porous, microstructured flat membranes. A custom designed mold allowed the formation of structures of defined shape and size. Figure 2 shows flat PES/PVP membranes with microwells of excellent quality. The microwells are homogenously distributed over the membrane, reflecting the features of the mold used for casting. Each well has 500 µm diameter and 400 µm depth in order to fit a range of pancreatic islets^18^. The cross-section of the membrane presents asymmetric pore morphology. A dense selective layer containing small pores (1–3 µm) is present on the bottom of the microwell membrane and provides protection from cell infiltration towards the microwells. A finger-like, porous sub-layer, with pores of less than 10 µm, is formed between the microwell structures and the top of the selective layer (Fig. 2B). This microwell array allows for good separation of the islets, preventing aggregation and spreading which could cause further transport limitation^14^. The islets do not adhere to the PES-based membranes due to its tailored hydrophilicity by addition of PVP. Therefore, their rounded morphology is preserved after prolonged culture (Fig. 2C and D). The number of the microwells determine the number of the islets possible for encapsulation in order to maintain their viability and function. Higher number of islets than the number of available wells used for encapsulation leads to transport limitation, thereby decreasing the functionality of the islets. Here, considering the number of wells, we could encapsulate 150 islets using 8 mm microwell membrane.

**Figure 3:**
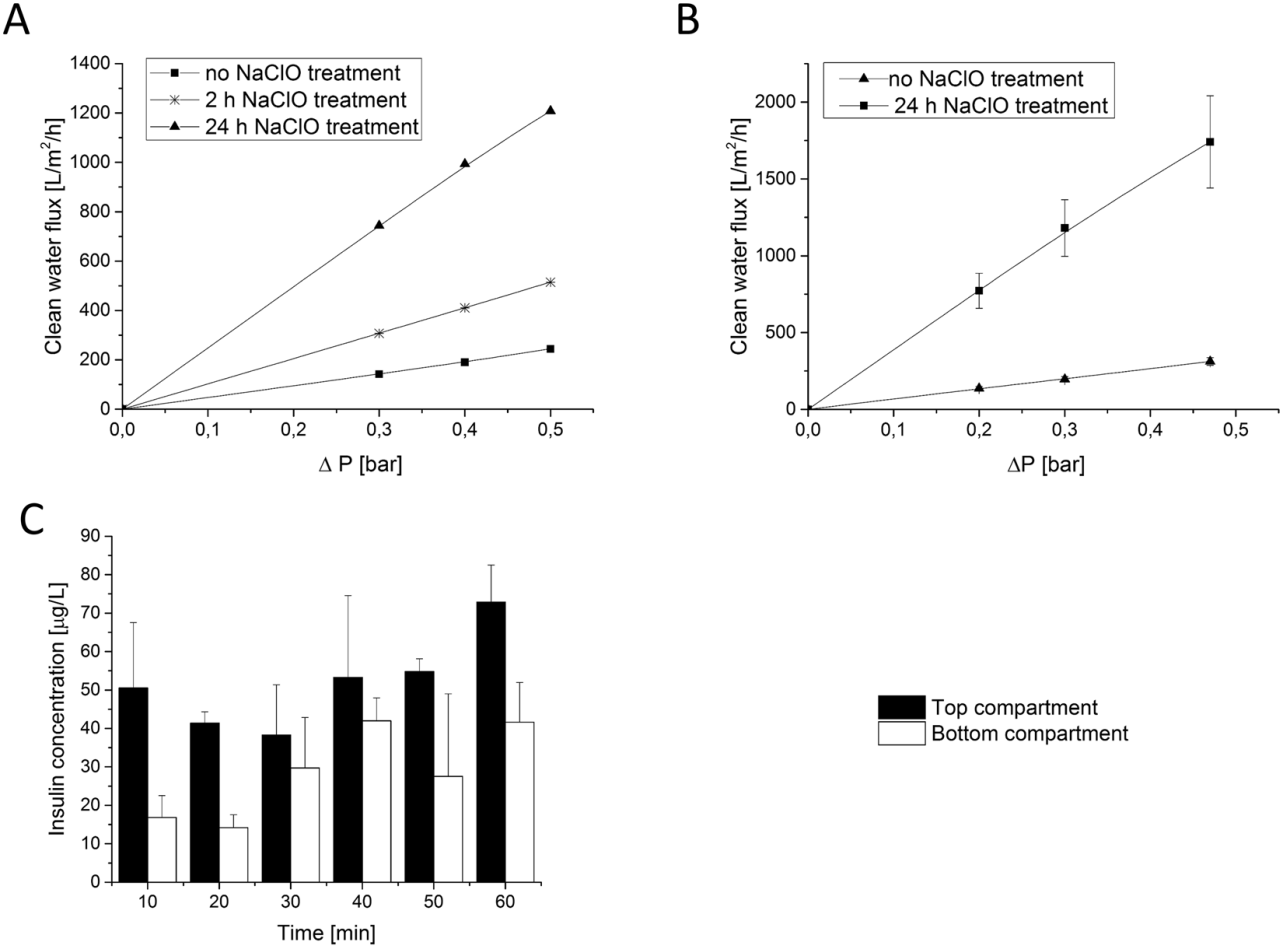
Transport characteristics. (**A**) Clean water flux vs. pressure for 250 µm thick membranes, (**B**) Clean water flux vs. pressure for 100 µm thick membranes, (**C**) MIN6 aggregates insulin secretion through the membrane in response to a high glucose concentration. Error bars indicate standard deviation (n = 3).

Figure 3A and B present the clean water fluxes (CWF) of the membranes at various transmembrane pressures. In all cases, the graph is linear indicating high stability of the membranes in this pressure range. Table 1 presents the water hydraulic permeability of all prepared membranes. When reducing the membrane thickness from 250 to 100 µm, the hydraulic permeability increases from 479 to 668 L/m^2^/h/bar. Additionally, treatment with NaClO solution known for partial removal of PVP from the membrane pores further improves membrane permeability. In fact, the hydraulic permeability of the membranes treated with NaClO for two hours is more than double compared to the untreated membrane. Longer treatment, namely for 24 hours, results in even higher hydraulic permeability of 3845 L/m^2^/h/bar.

**Table 1:** Membrane water permeability.

Microwell membrane	Membrane thickness [µm]	Sodium hypochlorite treatment [h]	Water hydraulic permeability (n = 3) [L/m^2^/h/bar]
M1	250	—	479 ± 9
M2	250	2	1020 ± 14
M3	250	24	2461 ± 39
M4	100	—	668 ± 17
M5	100	24	3845 ± 33

The important requirement for the microwell membrane to be suitable for islet encapsulation is having high insulin and glucose permeability. To test this, MIN6 aggregates consisting of 250 cells per aggregate were seeded in the microwell membranes assembled in the transwell system and exposed to 16.7 mM glucose solution in the bottom compartment. Over time, glucose diffusing through the membrane to the top compartment induces insulin secretion from the aggregates. Figure 3C shows that within 10 min the aggregates release insulin, which diffuses through the membrane to the bottom compartment. After one hour, the insulin concentration in the bottom compartment increases further. Based on these data, the estimated diffusion coefficients of insulin and glucose through the membranes are 0.3 × 10^–10^ and 3.6 × 10^−10^ m^2^/s respectively and they are in the same order of magnitude as the free diffusion coefficient of the molecules in solution, namely 1.5 × 10^−10^ m^2^/s for insulin and 9.59 × 10^−10^ m^2^/s for glucose. These results indicate that the porosity of microwell membranes is sufficient to achieve high transport of insulin and glucose without transport limitations.

In summary, we successfully fabricated microwell membranes suitable for our encapsulation device. The membrane performance was assessed further regarding the functionality of the pancreatic beta cell line MIN6 aggregates in an open system.

### MIN6 aggregates function using open system

**Figure 4:**
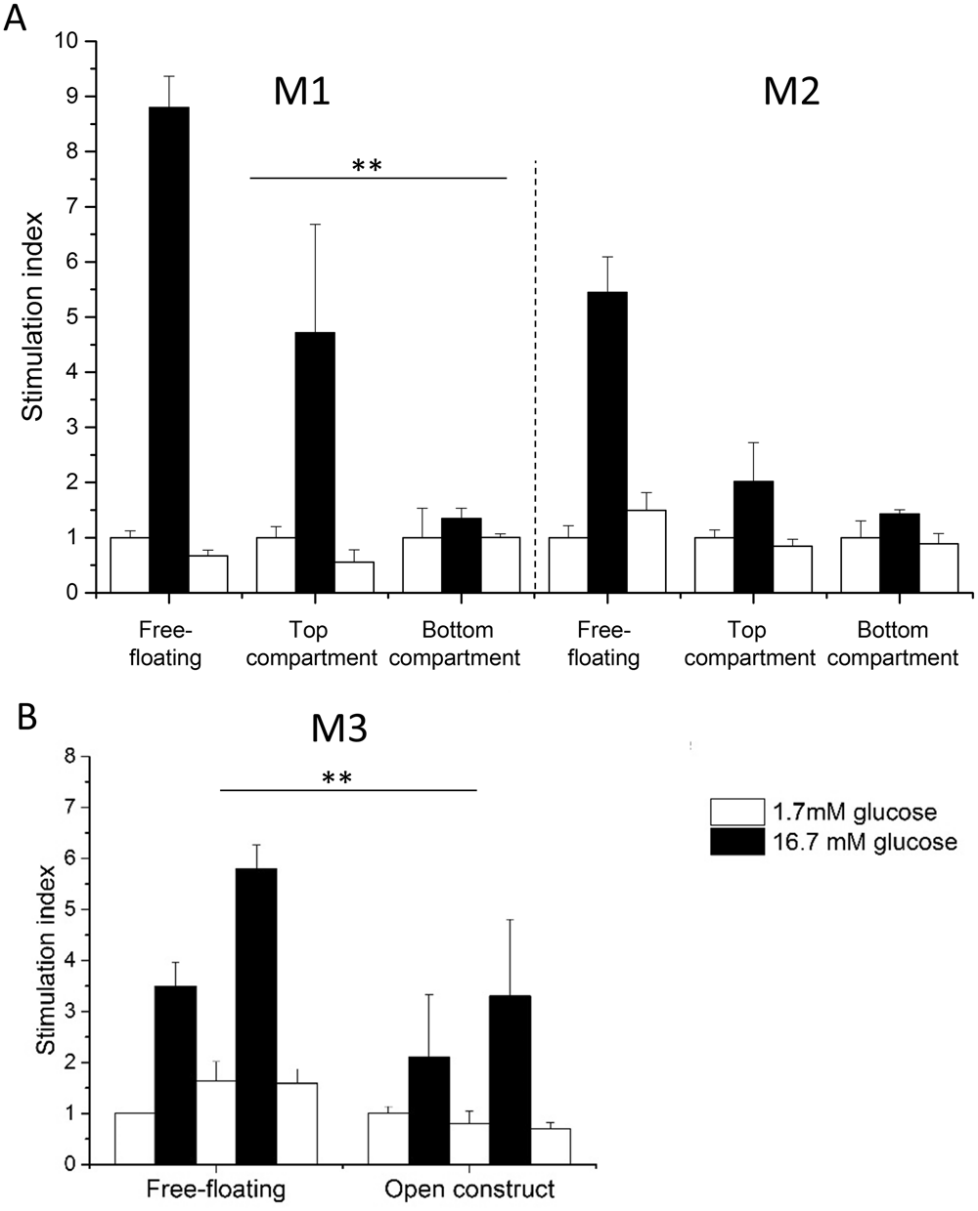
MIN6 aggregates functionality. (**A**) A comparison of MIN6 aggregates insulin secretion detected on the top and bottom compartment separately, in an open transwell system using untreated microwell membranes - M1 and microwell membranes after 2 h treatment with NaClO solution - M2, (**B**) Total insulin secretion of MIN6 aggregates over 5 h using final open construct with microwell membranes after 24 h of treatment with NaClO solution - M3; Insulin secretion is normalized to the first low glucose stimulation and presented as a stimulation index. Error bars indicate standard deviation (n = 3), **p < 0.05.

A glucose induced insulin secretion test (GIIST) was performed for the MIN6 aggregates seeded on the microwell membranes placed in the transwell system. Figure 4 compares the stimulation index of the aggregates within the microwell membranes to the free-floating aggregates, our positive control. For the estimation of the stimulation index, the insulin secretion of all samples was normalized to the insulin secretion of the first experiment of low glucose stimulation. Therefore, in all results presented here, the stimulation index of the first low is always equal to one.

In all cases, free-floating aggregates function well (stimulation index more than 2 for high glucose concentration) and show a clear response to glucose concentration changes. The insulin concentration was analyzed separately on the top and bottom compartments of the open microwell system with M1 and M2 membranes (Fig. 4A). The MIN6 aggregates on the top compartment respond to glucose concentration changes. Additionally, when more open, M2 membrane is used, the insulin concentration in the bottom compartment reaches a similar value to the one detected in the top compartment, where cells were in direct contact with the high glucose concentration solution. Finally, a function test of MIN6 aggregates over 5 h was performed in an open system, using the most permeable, 250 µm thick membranes – M3 (Fig. 4B). An increase in insulin release following stimulation, compared to basal insulin release levels, was observed there, although statistically different than the response of the free-floating aggregates.

In summary, we showed here the development of an optimized functional microwell membrane open system where the MIN6 aggregates within the wells respond to glucose concentration changes. The M3 membrane will be investigated in the next section as part of a closed system for human islet encapsulation.

### Human islet viability and functionality using closed system

**Figure 5:**
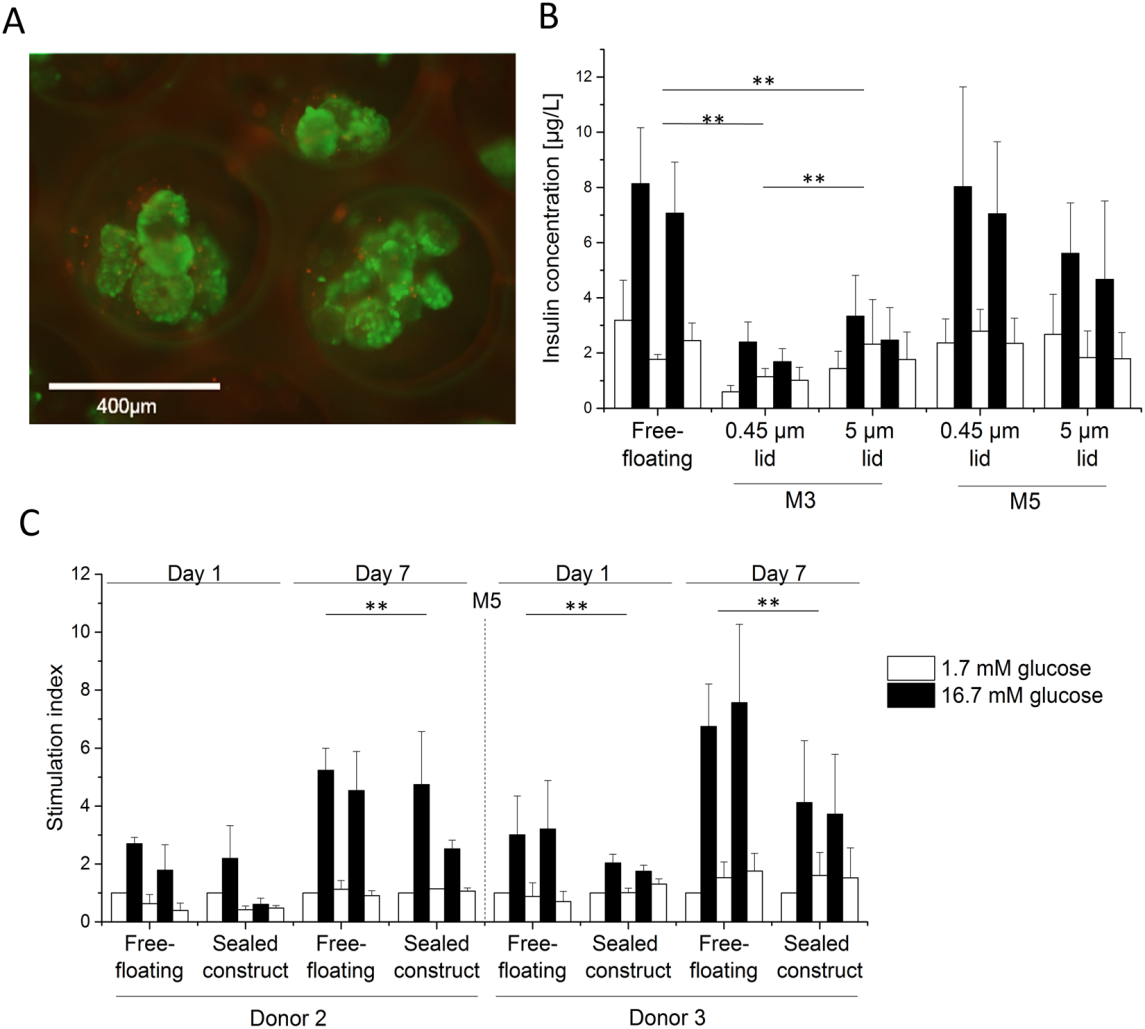
Human islets encapsulation. (**A**) Islets viability after 1 day of culture in closed system with M3 microwell membrane, green-live cells, red-dead cells, (**B**) Islets functionality using a closed system with various porosities of the lid and various thickness of microwell membranes: 250 µm thick microwell membrane after 24 h treatment – M3 and 100 µm thick microwell membrane after 24 h treatment – M5, (**C**) Functionality of islets from two donors over 7 days using a sealed device. To compare response to glucose concentration changes of islets from two donors, the insulin secretion is normalized to the first low glucose stimulation and presented as a stimulation index. Error bars indicate standard deviation (n = 3), **p < 0.05.

Human islets were seeded in the closed system with the M3 microwell membrane and cell survival was studied after one day of culture. Figure 5A shows that the islets are viable in the closed system, as represented by the green viable cells. As all the encapsulated islets were distributed between the wells and their size was smaller than the diameter of the well, designed to fit islets up to 500 µm, we observed in some wells small groups consisting few islets. However, these islets could freely move within the wells indicating that their aggregation was avoided.

We also investigated the islet function in our closed system consisting of microwell membrane covered with a flat PES membrane as a lid. We compared flat membranes with various pore sizes used on the top of the most permeable 250 µm (M3) and 100 µm (M5) thick microwell membranes. Figure 5B compares the insulin concentration obtained after glucose stimulation secreted from free-floating islets and islets encapsulated in the closed system. Both free-floating and encapsulated islets respond to glucose concentration changes. Islets encapsulated in closed system with the thin M5 microwell membrane respond over 5 h in similar manner to free-floating islets regardless of the porosity of the lid. However, for the thicker, M3, microwell membrane a lid with pore size of 5 µm is required to achieve sufficient glucose transport and obtain better insulin secretion, although significantly lower than for free-floating islets (p < 0.05). Since a membrane lid with 0.4–0.45 µm pore size is expected to provide sufficient barrier between the islets and recipient’s tissue from the top of the construct^19, 20^, we have selected this lid in combination with the 100 µm thick microwell membrane (M5) for the preparation of the sealed device. Figure 5C shows the stimulation index for the islets encapsulated there in comparison to free-floating islets from two different donors. The sealed islets produce and release insulin upon stimulation after one day of culture and they remain functional, indicating also their viability after 7 days of culture. Interestingly, during the 7 days’ culture period, they show an increase in performance comparable to the increase observed for the free-floating islets. The encapsulated islets respond to glucose concentration changes independent on the donor variability, although they secrete significantly lower amount of insulin in comparison to free-floating islets (Supplemental Figure 1) (p < 0.05).

## Discussion

In this study, we propose a novel membrane-based islet macroencapsulation device. We developed a sealed device that consists of a microwell membrane for hosting the islets and a flat membrane as a lid. Our device was developed using non-degradable polymer blend of PES/PVP^15, 21^. PES is an excellent membrane forming material with very good chemical and mechanical properties therefore it has been widely used in medical devices, artificial organs and blood purification processes, such as hemodialysis membranes^22–25^. PVP forms uniform blend with PES due to strong donor/acceptor interaction between O=CN functional groups from PVP and O=S=O from the benzene ring during membrane formation. By blending PES with PVP, we can obtain a more hydrophilic material with better biocompatibility^26, 27^, low fouling and non-cell adhesive properties^28, 29^. All these properties are essential for preventing islet clustering and attachment, and increase islet survival after transplantation^18, 30, 31^.

The microwell membranes were fabricated using PSµM method^15, 21^. Through immersion precipitation of the polymer on a micropatterned mold, we obtained in one step a highly porous material with controllable micrometer-scale pores having excellent quality microwells (see Fig. 4). Recently, Buitinga *et al*.^14^ developed an open poly(ethylene oxide terephthalate)-poly(butylene terephthalate) (PEOT/PBT) microwell scaffold fabricated by microthermoforming, where heated polymer porous film was stretched into a negative mold in order to obtain similar microwells. The microthermoforming method, however, affects pore morphology causing stretching and collapsing thus can have a negative effect on transport properties. Important advantages of PSµM applied here is the fabrication of a porous microstructured membrane in one step and the easy upscaling. The fabrication parameters can be tuned to control material shrinkage and obtain the suitable pore size, adequate porosity and interconnectivity of the microwells membranes, all very important for sufficient transport of nutrients to the encapsulated islets. In our device, the membrane porosity is carefully tailored to allow glucose diffusion to the islet and corresponding insulin release in response to blood glucose levels. The selective layer of the microwel membrane is microporous (1–3 µm) and therefore is expected to block the immune cells (size of ~10 μm^12^) and can provide sufficient immune isolation for encapsulated islets (see supplemental Fig. 2). Additionally, the device avoids aggregation of the islets, since they are seeded on separated microwells. According to the modeling studies of Dulong and Legallais^32^, in order to increase the number of functional islets, a higher islet density needs to be used. However, high islet density with no cell aggregation would require the application of a gel system to keep the islets separated leading to the need of large devices, almost impossible to apply in humans. Our microwell device prevents islet aggregation thereby improving their chances of survival and function, since separation allows for a proper supply of nutrients and oxygen to all islets. The dimensions of the microwells (500 × 400 um) are suitable for fitting the broad range of pancreatic islets obtained after islet isolation. Lehmann *et al*.^18^ describe the superiority of small islets over large ones due to higher survival in both normoxic and hypoxic conditions and better insulin secretion indicating that optimal mass transport plays an important role. Islets with a diameter around 150 µm are recommended for encapsulation in order to avoid necrosis, which usually occurs in larger islets^33^. Using our fabrication method, it is possible to create membranes containing microwells with smaller dimensions to accommodate a population of smaller diameter islets, or pseudoislets created from stem cell derived *de novo* beta cells, thereby further increasing beta cell survival and function. This is one of the main goals of a follow up study.

Clark *et al*.^34^ proposed heat-sealing as an effective method to close polysulfone hollow fiber membranes in order to prevent cell infiltration. Here, we applied heat sealing only on the edges of the membranes to prevent surface damage, and preserve pore morphology and porosity, which are important for adequate transport properties. Our device also features a practical small inlet, which makes islet seeding quite simple. After seeding, the islets settle on the bottom of the wells and remain stable during further handling procedures.

The pore size of the lid membrane is 0.45 µm, in agreement with other studies, which have shown that this pore size does not allow host cells to permeate to the device providing protection to allogeneic and xenogeneic transplants^19, 20, 35, 36^. For comparison, the TheraCyte^TM^ system, is composed of a cell impermeable 0.4 µm pore membrane laminated to a 5 µm outer membrane for support and tissue integration^12^. Besides, Cell Pouch System^TM^ - a biocompatible polymeric macrocapsule, mimicking natural environment in the host for encapsulated pancreatic cells contains large pores to allow the development of fibrous tissue rich in vessels without immunoprotection whereas the “Islet sheet” macroencapsulation system consists of flat, thin alginate sheets in which islets are entrapped without efficient islet separation^37, 38^.

In order to evaluate the function of our device, we used MIN6 insulin secreting cells, which are able to create stable aggregates and mimic pancreatic islet function^39^. These aggregates function when seeded in the open 250 µm thick microwell membranes (M1). By tuning the membrane porosity (via partly washing PVP with NaClO) we obtained membranes with optimal diffusion of insulin (M2). In fact, the concentration of insulin, which passes through the membrane equalizes after 30 minutes to the one above the membrane, where cells in the microwells are in direct contact with the glucose solution, showing that insulin secreted by the beta cells can be transported relatively unhindered across the membrane. When using microwell membranes with 5 times higher water permeability (M3) than the original membrane (M1), pseudo-islets seeded in the membrane functioned well (stimulation index above 2), although the stimulation indexes were lower than in case of free-floating aggregates. Wienk *et al*.^40^ first reported an increase in water permeability of PES/PVP membranes attributed to PVP degradation and leaching during membrane treatment with a NaClO solution. This observation was also later confirmed by other researchers^28, 41, 42^. Here, the membrane treatment with NaClO allowed increase of membrane porosity and optimization of the membrane transport properties, however, at the same time not all PVP was removed so the membranes have still low fouling and low adhesive properties (see Fig. 4).

Islets encapsulated in our closed system using 100 µm thick microwell membrane (hydraulic permeability 3845 [L/m^2^/h/bar]) maintain their glucose responsiveness comparable to free-floating islets, regardless of the porosity of the membrane lid. This fits well with the results of literature studies which indicate that when the diffusion distance of cells to nutrients is lower than 200 μm, cell survival could be improved^12, 43^. Besides, our results confirm that decreasing membrane thickness, thereby reducing the distance that molecules such as glucose and insulin would need to travel through the membrane, has also a positive effect on islet functionality. Moreover, islets response to glucose concentration changes was improved after longer culture for 7 days (stimulation index 2 times higher for high glucose stimulation in comparison to day 1). Due to isolation and handling procedure islets are exposed to cellular stresses, therefore their functionality might be affected during first day of *in vitro* culture. However islet culture for longer period allows for their recovery and results in improved their functionality.

In comparison to other devices reported in the literature, our device combines two important characteristics: it avoids aggregation of the islets, since they are seeded on separated microwells, and is expected to protect them from the host immune cells via the tailored membrane porosity. It is finally important to note here that the developed membranes are mechanically stable and all the above steps can be performed without problems. Preliminary implantation studies in mice (results not shown) indicated that the device can be easily implanted and retrieved.

## Conclusions and Outlook

In this study, we have developed a novel PES/PVP device for macroencapsulation, in which islets are physically separated in microwells and closed by a membrane lid, without compromising their function. Non-degradable PES/PVP membranes are mechanically stable and can offer long-term protection of encapsulated islets. Moreover, low adhesive material properties combined with our specific microwell design prevent islet spreading and aggregation. Additionally, the tailored membrane porosity allows for sufficient glucose and insulin transport, crucial for maintaining islet viability and function.

This study showed the proof of concept of applying microwells membranes for islets encapsulation. We designed membranes with microwells of 500 µm in diameter in order to fit the broad size range of islets available for us. Since for clinical implementation, small size islets (50–150 μm) which show higher viability and function comparing to bigger islets^18^ should be used, in the future, we will fabricate membranes with smaller wells (diameter of 200 µm) and with smaller spacing between the wells (50 µm). It is generally perceived that around 540000 islets should be sufficient to restore normoglycemia in a patient of 70 kilograms^44^. Based on this, we estimate that we would need 4 of those membranes with diameter of 10 cm in order to encapsulate clinically relevant number of islets. Besides, the thin film design of our device allows the creation of multilayer stacks of microwell membranes similar to those developed in our laboratory recently for upscaling of tissue engineering constructs^45^, enabling possibly the development of one compact microwell device.

## Materials and Methods

### Microwell membrane fabrication

**Figure 6:**
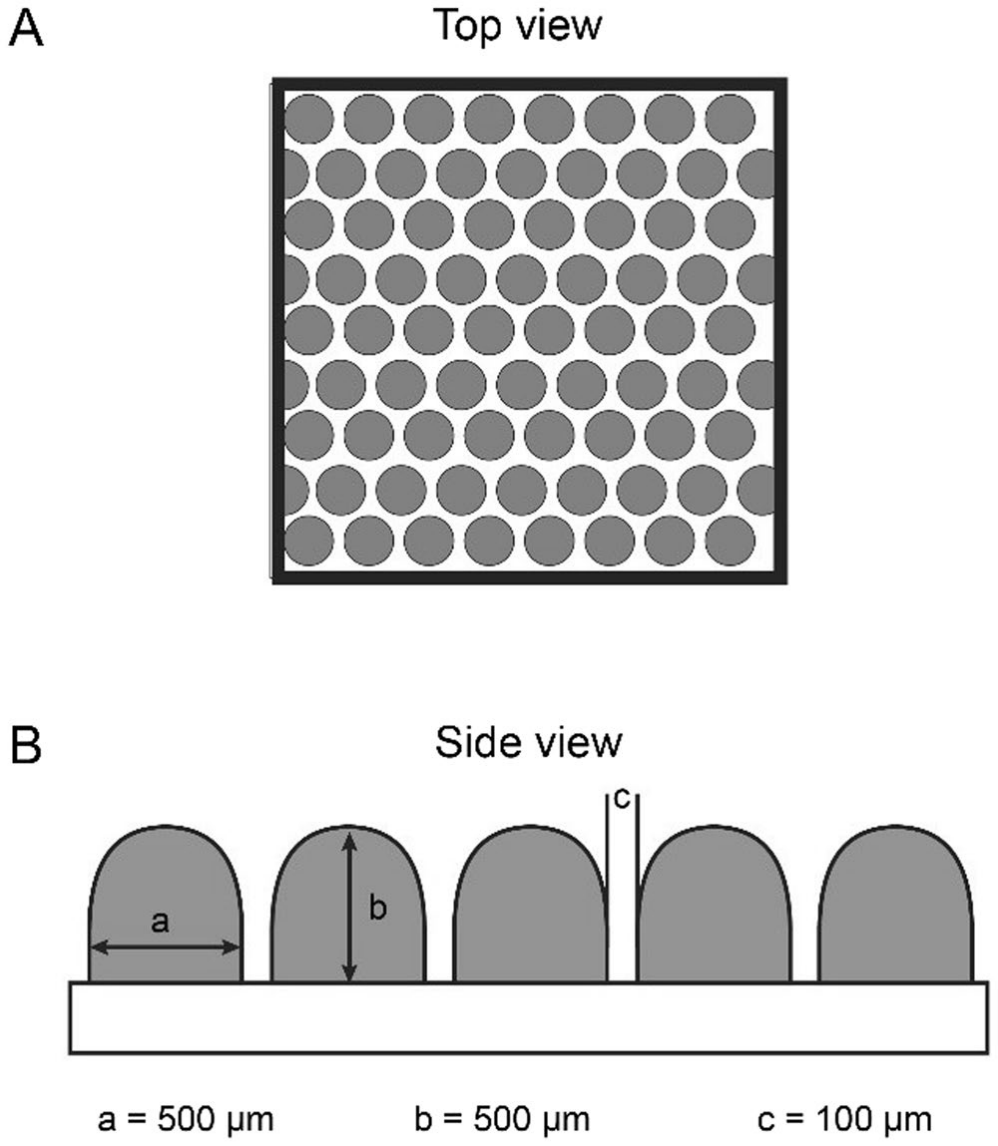
Silicon micropatterned mold. (**A**) Top view. (**B**) Side view.

Microwell membranes were fabricated using phase separation micromolding (PSµM)^15–17^. For this, we used a polymer blend of 15 wt.% poly(ethersulfone) (PES) (Ultrason, E6020P) and 5 wt.% polyvinylpyrrolidone (PVP) (MW = 40000, Sigma Aldrich) in N-Methylpyrrolidone (NMP) (Acros organic). This polymer solution was stirred on a roller bank overnight, at room temperature. The membranes were prepared by casting the solution on a custom made, silicon, micropatterned mold with spatially organized dome-like structures of 500 µm height and 500 µm in diameter presented on Fig. 6.

A custom-made casting machine, with micrometric screws to regulate the casting thickness, was used to obtain 100 µm and 250 µm thick membranes. Casting was followed by immersion into a coagulation bath, containing demineralized water. After the polymer solution became turbid and precipitated, the membranes were removed from the mold, rinsed with demineralized water in order to remove remaining solvent traces and stored in demineralized water till further use. In order to increase the membrane porosity, the membranes were treated with 4000 ppm sodium hypochlorite aqueous solution (NaClO, Fluka) for either 2 h or 24 h. Subsequently, the membranes were washed and stored in demineralized water.

### Scanning electron microscopy

For scanning electron microscopy (SEM), the membranes were dried overnight in air at room temperature and cryogenically broken in liquid nitrogen when needed for cross section images.

Microwell membranes with cells were fixed in 4% paraformaldehyde for 1 h at room temperature, dehydrated in water-ethanol solutions following a gradient (volume ratio water: ethanol of 100:0, 50:50, 25:75, 10:90, 5:95 and 0:100) and dried after dipping in hexamethyldisilizane overnight. Dried membranes were placed on the SEM holders and sputter-coated with nm-thick gold layer prior to imaging.

### Cell culture and controlled cell aggregate formation

MIN6-B1 mouse insulinoma cells (kindly provided by Dr. P. Halban, University Medical Center, Geneva, Switzerland) were cultured in Dulbecco’s Modified Eagle’s Medium (DMEM, Gibco) supplemented with 10% (v/v) FBS (Lonza), 100 U/mL penicillin and 100 mg/mL streptomycin (Gibco) and 70 µM freshly added beta-mercaptoethanol (Gibco) at 37 °C and 5% CO_2_.

Sterile agarose microwells were fabricated as described previously^46^. In short, polydimethylsiloxane (PDMS) negative molds carrying 200 µm pillars were sterilized with 70% ethanol. 3% UltraPure^TM^ agarose (Gibco) was dissolved in PBS. The solution was heated to 100 °C in a microwave oven. Molds were placed inside a 6-well plate and filled with 8 mL of 3% agarose solution. The plates were centrifuged at 300 g for 1 min to remove air bubbles and stored at 4 °C for at least 30 min. After the gel was formed, the molds were gently removed from the agarose using a sterile spatula. Using a sterile punching device, chips were punched out leaving a thin agarose wall on all sides to fit into a 12-well plate. Stable pseudo-islets were then prepared based on the work of Hilderink *et al*.^47^: MIN6 cells were then seeded onto the agarose chips (250 cells per pseudo-islet). The plates were centrifuged at 150 g for 1 min and 2 mL of medium was carefully added to the chips. Medium was refreshed 24 h after seeding. After 48 h at 37 °C, aggregates (80–100 µm in diameter) were flushed out of the chips and used for seeding on the microwell structured membranes.

Human islets of Langerhans isolated from 3 donor pancreata (purity 85%, 85% and 75% respectively) were provided by the Human Islet Isolation Laboratory at the Leiden University Medical Center (Leiden, The Netherlands). Studies were only performed on islets that could not be used for clinical transplantation and for which research consent was available, according to national laws. The islets were cultured in CMRL 1066 medium (5.5 mmol/L glucose) containing 10% FBS, 2 mM GlutaMAX, 100 mU/mL penicillin and 1 mg/mL streptomycin (Gibco), 10 mmol/L HEPES, and 1.2 mg/mL nicotinamide.

### Membrane transport properties: water permeability and insulin, glucose diffusion

Microwell membranes with an effective surface area of 0.9 cm^2^ were used for clean water flux measurements. The experiments were performed at room temperature using nitrogen pressurized dead-end ‘Amicon type’ ultrafiltration cell and MiliQ water. Firstly, the membranes were pre-pressurized for 30 minutes at 0.7 bar. Afterwards, the clean water flux through the membranes at various transmembrane pressures was measured for at least 20 minutes. The membrane hydraulic permeability was calculated from the slope of the linear part of the flux versus the transmembrane pressure relation.

**Figure 7:**
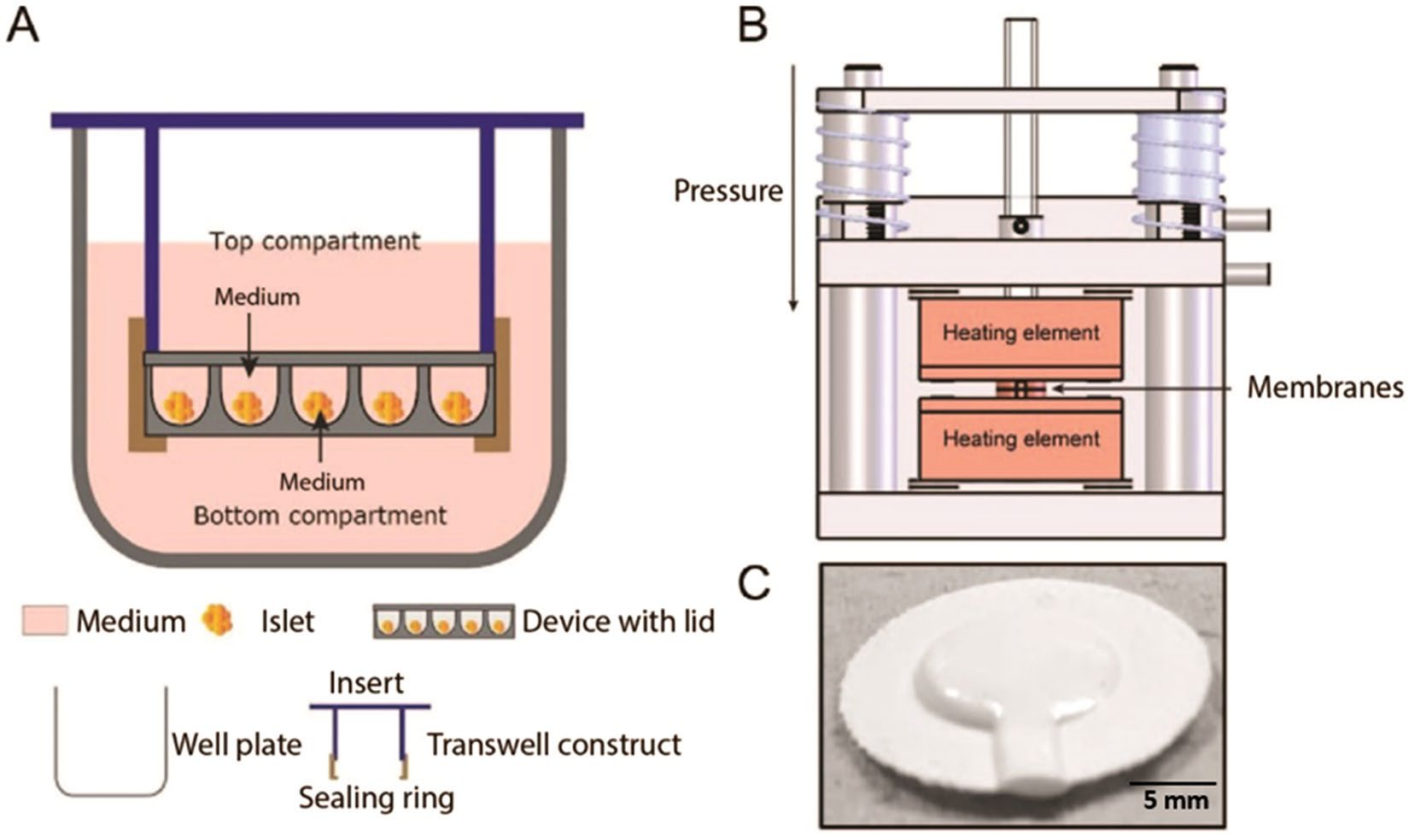
Experimental setup. (**A**) Schematic representation of two-compartment transwell system, (**B**) Schematic overview of the custom made sealing machine. Two Teflon coated heating elements with a circle with a small opening were used to obtain a seal between the microwell membrane and the PES lid, (**C**) The sealed device.

In order to determine the diffusion coefficient of insulin and glucose through the microwell membranes, a two-compartment transwell system was used. The commercial membrane was removed from the Transwell insert (Corning) and a ‘sealing’ poly ether ether ketone (PEEK) ring was fabricated to seal the microwell membrane to the insert (Fig. 7A). The assembled Transwell insert was placed in the well plate, creating top and bottom compartments separated by the microwell membrane. The membranes were sterilized in 70% ethanol, washed with PBS and pre-incubated in culture medium. Modified Krebs buffer (115 mM NaCl, 5 mM KCl, 24 mM NaHCO_3,_ Sigma) supplemented with 2.2 mM CaCl_2_, 20 mM HEPES (Gibco), 30% bovine serum albumin, 1 mM MgCl_2_, and 0.1 mM Theophylline (Sigma) was prepared at pH 7.4^48^. From this Krebs buffer, a high (16.7 mM) glucose solution was prepared. For the glucose diffusion experiment, the top compartment of the transwell system contained the prepared buffer, whereas the bottom compartment contained glucose solution. In the case of insulin diffusion experiment, a 50 µg/L insulin in glucose solution was prepared and put in the top compartment, while the bottom compartment contained the earlier prepared buffer. After 1 hour, samples were collected separately from both compartments (n = 6).

In order to evaluate whether the membrane limits the response to glucose concentration, 150 MIN6 pseudo islets were seeded on the top of the microwell membrane. Subsequently, the medium was replaced by the premade buffer on the top compartment and glucose solution on the bottom compartment. In response to glucose diffusing through the membrane to the top compartment, the MIN6 aggregates there secreted insulin. During a period of 1 hour, samples were taken separately from the top and bottom compartment every 10 minutes. The samples were analyzed for glucose concentration using a Vitros DT60 chemistry system and insulin concentration using a Mouse ELISA immunoassay (Mercodia). The amount of glucose and insulin that passes through the membrane, from the donor compartment to the acceptor compartment, in time, indicates whether the porosity of the membrane is optimal for transport of these molecules. The permeability of glucose and insulin through the membranes was calculated using the equation:

1 $$permeability\,({m}^{2}\,{s}^{-1})=\frac{flux(g{m}^{-2}\,{s}^{-1})}{{\rm{\Delta }}C\,(g\,{m}^{-3})}\times l\,(m)$$

where, ΔC is the concentration difference between the donor and the acceptor compartment and l is the geometrical thickness of the membrane. Flux was calculated using the equation:

2 $$flux\,(g{m}^{-2}{s}^{-1})=\frac{[{C}_{acceptor}(g{m}^{-3})x{V}_{acceptor}({m}^{3})/A\,({m}^{2})]}{time\,(s)}$$

where, C_acceptor_ is the glucose or insulin concentration in the acceptor compartment, V_acceptor_ is the volume of the acceptor compartment and A is the surface area of the membrane. From the permeability coefficient, we calculated the diffusion coefficient using equation 3:

3 $$diffusion\,coefficient\,({m}^{2}{s}^{-1})=\frac{permeability\,({m}^{2}\,{s}^{-1})}{K}$$

where K is the partition coefficient of glucose and insulin in the membrane, which was considered to be equal to one.

### MIN6 aggregates functionality *in vitro* using open system

The membranes for cell seeding were placed in the transwell system (Fig. 3A), sterilized with 70% ethanol for 30 min and washed 3 times in PBS. 150 MIN6 aggregates in 300 µl of medium were seeded on the top of the microwell membranes and 300 µl of medium was added to the bottom compartment. After 1 day of culture, a glucose induced insulin secretion test (GIIST) was performed, with free-floating pseudo-islets in a commercial transwell system (MilliPore), as a control. The modified Krebs buffer was prepared as previously described and was used to prepare low (1.67 mM) and high (16.7 mM) glucose concentration solutions. Both the free-floating pseudo-islets and the microwell membranes with MIN6 aggregates were washed three times (5 min) in the Krebs buffer, followed by a pre-incubation of 90 min in the low glucose concentration buffer. All samples were then incubated for 60 min in subsequent low, high and low glucose concentration buffer with three times 5 min washing in the Krebs buffer, between each high and low. The final function test was performed with additional high and low glucose concentration buffer incubation. During the test, both top and bottom compartments of the transwell system contained the same buffer for each step respectively. Samples were taken after each incubation time, spun down (300 g, 3 min) and the supernatant was stored at −20 °C. Samples were analyzed using insulin Mouse ELISA immunoassay (Mercodia) according to the manufacturer’s instructions. The functionality of MIN6 aggregates was determined by the stimulation index that is defined as the insulin secretion when stimulated with the first low glucose buffer.

### Human islets viability in closed system

The closed microwell system (see details about its preparation in the next section), containing 150 human islets, was opened after 1 day of culture and live/dead analysis was performed in order to examine cell viability. Membranes with islets inside the wells were placed in a solution containing 0.25 µl/mL calcein (green) and 3 µl/mL ethidium homodimer (red) in PBS and incubated for 30 min at 37 °C in the dark. Green-fluorescent (ex 494 nm/em 517 nm) live cells and red florescent (ex 517 nm/em 617 nm) dead cells were imaged using an EVOS digital inverted fluorescence microscope and photomicrographs were taken.

### Human islets functionality *in vitro*

#### Closed system

A closed system containing a microwell membrane covered with a flat PES membrane lid was developed. For the lid, we investigated membranes with two different porosities (0.45 µm and 5 µm, Sterlitech). These membranes were sterilized with 70% ethanol for 30 min, washed 3 times in PBS and pre-incubated in culture medium overnight. Afterwards, the microwell membrane was assembled within the transwell system and human islets (150) in 300 µl of medium where seeded on the top. Medium was carefully aspirated, leaving the islets inside the wells of the membrane. The insert was removed while the microwell membrane with islets remained within the sealing ring. The lid was placed on the top of the microwell membrane and the system was sealed with the insert fitted in the sealing ring.

#### Sealed device

A sealed device was designed with the aim of implantation. The microwell membrane and the flat lid membrane were placed between the two shaped molds of a custom-made sealing machine (Fig. 7B,C). A temperature of 90 °C was applied for 10 seconds and the membranes were sealed on the edges, leaving open the middle part and a small inlet for cell seeding. The sealed device was sterilized with 70% ethanol, washed in PBS and pre-incubated in culture medium overnight. The islets in 10 µl of medium were seeded inside the device via the small inlet, which was closed after seeding using sterile, surgical staples (Teleflex Medical, HORIZON, Ligating clips).

To assess the encapsulated islets’ function, a glucose induced insulin secretion test (GIIST) was performed after culturing them statically for 1 day and 7 days. Free-floating islets in transwell system (MilliPore) (n = 3) were used as a control. Islets were first pre-incubated in modified Krebs buffer (115 mM NaCl, 5 mM KCl, 24 mM NaHCO_3_, 2.2 mM CaCl_2_, 20 mM HEPES, 1 mM MgCl_2_, 2 mg/mL bovine serum albumin, pH 7.4) for 90 min at 37◦C and 5% CO_2_. All samples were then stimulated for 1 hour in subsequent low (1.67 mM), high (16.7 mM), low, and again high and low glucose buffer with three times 5 min washing in the Krebs buffer between the high and low glucose incubation step. Samples were taken after each incubation, spun down (300 g, 3 min) and the supernatant was stored at −20 °C. Samples were analyzed using an insulin ELISA (Mercodia). The functionality of human islets was determined by the stimulation index, which is defined as the insulin secretion after stimulation with high glucose buffer relative to the insulin secretion when stimulated with the first low glucose buffer. Islets with stimulation index higher than two were regarded functional.

### Statistical analysis

Results are presented as the mean ± standard deviation. Statistical analyses were performed using two-tailed analysis of variance (ANOVA) using SPSS Statistics software (version 24, IBM Corporation) to compare the insulin concentration and stimulation indexes upon glucose stimulation for MIN6 aggregate and islets seeded within various microwell membranes. Statistical significance was considered at p- values < 0.05.

### Data availability

The datasets generated and analyzed during the current study are available from the corresponding author on reasonable request.

## Electronic supplementary material

Supplement information
