# Supplementary material for: Pancreatic islet macroencapsulation using microwell porous membranes
Source: Sci Rep. 2017 Aug 23;7:9186. doi: 10.1038/s41598-017-09647-7 (PMC5569024; doi:10.1038/s41598-017-09647-7)
Supplement: Supplementary file 1 — Supplement information [file 41598_2017_9647_MOESM1_ESM.pdf]

Pancreatic islet macroencapsulation using microwell porous membranes

Katarzyna Skrzypek[a], Milou Groot Nibbelink[b], Jéré van Lente[b], Mijke Buitinga[c], Marten A. Engelse[d], Eelco J.P. de Koning[d,e], Marcel Karperien[b], Aart van Apeldoorn[b,f], Dimitrios Stamatialis[a] *

Author affiliation

[a]Bioartificial organs, Biomaterials Science and Technology department, MIRA Institute of Biomedical Technology and Technical Medicine, University of Twente, The Netherlands

[b]Developmental BioEngineering, MIRA Institute of Biomedical Technology and Technical Medicine, University of Twente, The Netherlands

[c]Department of Radiology and Nuclear Medicine, Radboud University Medical Center, Nijmegen, The Netherlands

[d]Nephrology, Leiden University Medical Center, The Netherlands

[e]Hubrecht institute, Utrecht, The Netherlands

[f]Complex Tissue Regeneration, MERLN Institute for Technology Inspired Regenerative Medicine, Maastricht University, The Netherlands

*Corresponding author:
Dimitrios Stamatialis
d.stamatialis@utwente.nl
P.O Box 217, 7500 AE Enschede, The Netherlands
tel: +31534892968

Supplemental material:

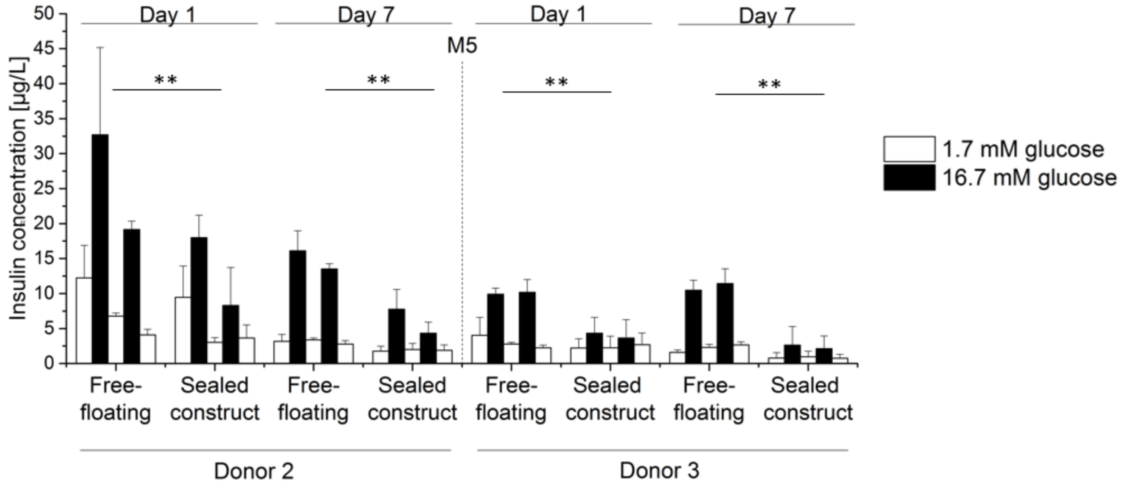

Supplemental figure 1. *Insulin secretion upon glucose stimulation of islets from two donors over 7 days using a sealed device. Error bars indicate standard deviation (n=3), **p<0.05.*

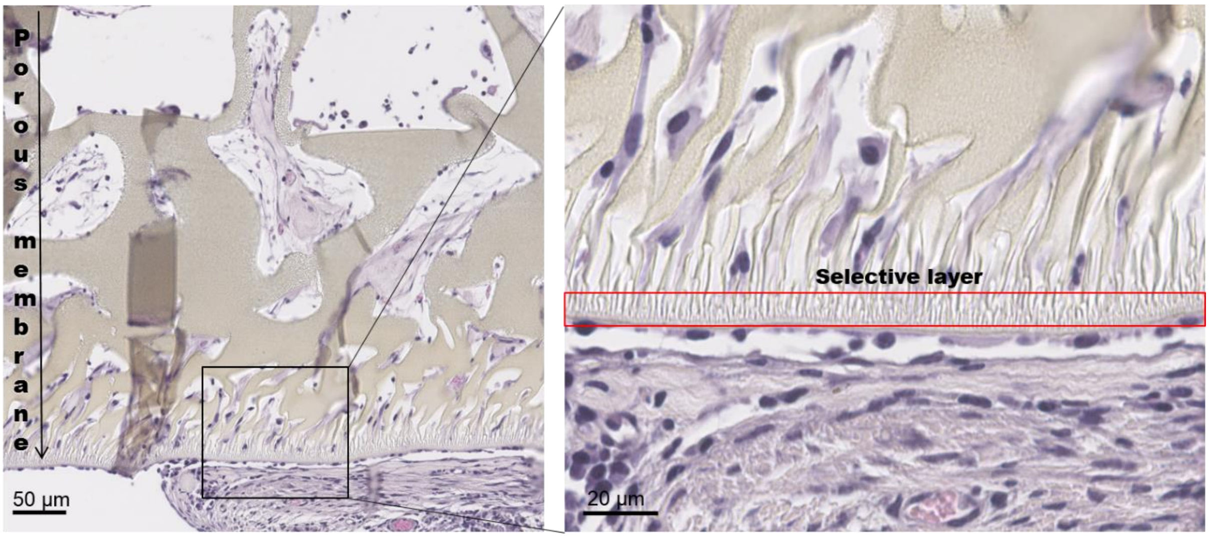

Supplemental figure 2*. Microwell membranes were implanted in epididymal fat pads of a mouse. After 28 days, samples were explanted, sectioned and stained with hematoxilin and eosin. A high magnification of the hematoxylin and eosin stained membrane section shows cell infiltration up to selective layer (on the left). The selective layer in more detail is indicated by the red outlined area (on the right).*
